# The Impacts of Low Diversity Sequence Data on Phylodynamic Inference during an Emerging Epidemic

**DOI:** 10.3390/v13010079

**Published:** 2021-01-08

**Authors:** Anthony Lam, Sebastian Duchene

**Affiliations:** Department of Microbiology and Immunology, Peter Doherty Institute for Infection and Immunity, University of Melbourne, Melbourne, VIC 3010, Australia; a.lam6@student.unimelb.edu.au

**Keywords:** phylodynamics, SARS-CoV-2, Bayesian phylogenetics, birth–death, coalescent

## Abstract

Phylodynamic inference is a pivotal tool in understanding transmission dynamics of viral outbreaks. These analyses are strongly guided by the input of an epidemiological model as well as sequence data that must contain sufficient intersequence variability in order to be informative. These criteria, however, may not be met during the early stages of an outbreak. Here we investigate the impact of low diversity sequence data on phylodynamic inference using the birth–death and coalescent exponential models. Through our simulation study, estimating the molecular evolutionary rate required enough sequence diversity and is an essential first step for any phylodynamic inference. Following this, the birth–death model outperforms the coalescent exponential model in estimating epidemiological parameters, when faced with low diversity sequence data due to explicitly exploiting the sampling times. In contrast, the coalescent model requires additional samples and therefore variability in sequence data before accurate estimates can be obtained. These findings were also supported through our empirical data analyses of an Australian and a New Zealand cluster outbreaks of SARS-CoV-2. Overall, the birth–death model is more robust when applied to datasets with low sequence diversity given sampling is specified and this should be considered for future viral outbreak investigations.

## 1. Introduction

Genomic surveillance of infectious diseases has enabled researchers to understand the transmissive behaviours of pathogen dynamics through phylodynamic inference. Since coining the term “phylodynamics” in 2004 [1], improvement in sequencing technology have allowed researchers to rapidly sequence unprecedented quantities of genetic data during the early stages of viral emergence. This in turn has allowed phylodynamic inference to give insight into epidemiological features such as the timing of emergence, population dynamics, and epidemiological parameters. Thus, providing valuable information to devise strategies to promptly mitigate the impacts of disease outbreaks. Bayesian inference has been valuable to this end. The key to this approach is that the data, via the likelihood is combined with prior information and a model to make epidemiological and evolutionary inferences. Assessing epidemiological models is critical for the uptake of phylodynamics in future disease outbreak investigations.

The branching process of the tree is determined by the phylodynamic model selected, also known as a “tree prior” and this ultimately affects the inference on the epidemiological parameters. The model selected must describe both the evolutionary and population dynamics of the genetic data [2]. In an outbreak context, the simplest model used is based on exponential growth of an infected population over time. Over the years, work has been done to incorporate more complex models for Bayesian phylogenetic inferences, i.e., models incorporating susceptible-infected-recovery status (SIR, SIS, and SI) and population structure [3,4]. The two most commonly utilised models in emerging infectious disease outbreak investigations are based on the coalescent and the birth–death process [3], which is the focus in our study.

In the early stages of epidemics, phylodynamic analyses provide insight to key epidemiological parameters by using the resulting tree from the sequencing data as a proxy to the incomplete transmission tree [5]. Depending on the settings, both the coalescent and birth–death models can effectively estimate exponential growth rate, r, which is the measure of infected exponential population size growth [6]. In turn, the growth-rate is the difference between the transmission rate, λ, and the become uninfectious rate, δ. The duration of infection is 1/δ, while λ is also known as the “birth” rate. Similarly, the basic reproductive number, R_0_, which is the average number of individuals an infected individual will infect in a fully susceptible population is estimated as R_0_ = λ/δ. In an epidemiological context, R_0_ is often used to measure the infectiousness of a disease. For example, R_0_ has been estimated at about 2.5 for Ebola virus, 1.1 for seasonal influenza, and around 2.5 for SARS-CoV-2 [7,8]. R_0_ is closely associated to r by r = λ − δ.

Although phylodynamic models can estimate key epidemiological parameters, the process for achieving these estimates differ. The coalescent model is typically defined as a deterministic population process, meaning the branching process is determined at any given time by a deterministic effective population size function [6], although approaches that relax the assumption of a deterministic population trajectory exist [9,10]. Whereas the birth–death model accounts for stochastic population size change, that explicitly models sampling using the sequence sampling times and the branching process is typically formulated forwards in time [11]. The birth–death model is parametrised with δ equating to the addition of recovery rate (μ) and sampling rate (ψ), (δ = μ + ψ) and sampling proportion (p) given as (p = ψ/(μ + ψ)). The birth–death and coalescent models can lead to inferences of the growth rate and R_0_ parameters, but the fact that they make different assumptions means that model and prior specification should be carefully considered.

Implementing the coalescent model is less complex than the birth–death (i.e., it typically does not have a sampling parameter) and this makes it a popular choice among these analyses. However, emerging epidemics involve stochastic population growth, which often needs to be accounted for when sampling proportion is high and population size is small, an important limitation of the deterministic nature of the population trajectory of the coalescent model [9]. On the other hand, the birth–death model can account for these limitations by modelling the sampling times from the sequence data [11]; however, the constant birth–death model assumes constant probability of sampling over time which may not be observed during disease outbreak scenarios. Irregular sampling that violates this assumption and is not specified in the model may lead to sampling bias resulting in inaccurate estimates of epidemiological dynamics [9]. If sampling bias is well understood, this problem can be alleviated by including variable sampling effort in the model, for example with a birth–death skyline model [12].

The term “phylodynamic threshold” refers to the required time for viruses to evolve such that reliable estimates of evolutionary rates can be drawn, a prerequisite for phylodynamic inferences [13]. In emerging disease outbreak investigations, often there is limited sequence data and lack of intersequence genetic variation, which may result in the tree prior driving the epidemiological estimates. Given the coalescent model is conditioned on sampling times, sequence data with low diversity (and low information content) may results in uncertain or even biased estimates of epidemiological parameters. In contrast, the birth–death model explicitly exploits sampling times, which may reduce the uncertainty in the epidemiological estimates [14], assuming that sampling is modelled correctly.

Here we investigate the impact of genetic variation between samples in acquiring reliable estimates of key epidemiological parameters for the coalescent exponential-growth and constant birth–death model. In our study, we compared the impact on model choice and the corresponding performance without the aid of highly informative priors on limited genetic data. Evaluating these models is crucial for future model uptake in emerging infectious disease outbreaks.

## 2. Materials and Methods

### 2.1. Simulation Study

Phylogenetic trees resembling an emerging SARS-CoV-2 outbreak were simulated under a birth-death process in MASTER v6.1.1 [15], with the parametrization λ = 2.5, δ = 1, p = 0.9, R_0_ = 2.5, r = 1.5 and the length of the process was stopped at 3 time units. These parameters were selected to represent an outbreak of SARS-CoV-2 using similar R_0_ estimations from previous studies and sampling probability of 0.9 to imitate a highly sampled cluster [16]. Our time units were defined as per duration of infection, so if we consider a SARS-CoV-2 outbreak, with a duration of infection of 10 days, then the process would have run for 30 days. This process was repeated five times to generate 5 phylogenetic trees and due to the stochastic nature of these simulations, each tree had varying number of tips (21, 38, 56, 82 and 129 tips). Recent SARS-CoV-2 literature reveals that the molecular clock is around 10^−3^ subs/site/year, and the duration of infection (1/δ) is approximately 10 days [17,18]. The molecular clock rate was parameterised to subs/site/duration of infection (subs/site/(1/δ)) instead of the standard subs/site/year.

To measure the effects of the amount of genetic variation on acquiring reliable estimates, three molecular clock rate settings were chosen and set to 0.01, 0.005/36.5, and 0.001/36.5. The 36.5 denominator was used to scale our clock rate relative to the become uninfectious rate of SARS-CoV-2, which is 36.5 years^−1^ (i.e., 1/δ = 10 days, such that δ = 365 days/year/10 days, or 36.5 years^−1^). The 0.01 setting represents a large accumulation of intersequence genetic variation, the 0.001/36.5 setting represents a medium evolutionary rate per duration of infection and 0.005/36.5 represents five times the medium rate. Note that if our duration of infection is 10 days, the clock rate of 0.001/36.5 would be equivalent to 1 × 10^−3^ subs/site/year, and therefore comparable to recent estimates of SARS-CoV-2 early in the pandemic [13,19]. For each molecular clock rate settings, we simulated sequence evolution along the five phylogenetic trees 10 times and sequence alignments of 29,000 nucleotides were then generated and the number of variable sites recorded using NELSI [20] and Ape 5.0 [21]. In total we had 5 trees with 3 different clock rates and 10 replicates of each, to generate a total of 150 simulated alignments.

Bayesian phylogenetic analyses were conducted on these simulated sequence datasets using the coalescent exponential and the birth–death model through BEAST2 v2.6.2 [22]. The analyses ran for a length of 5 × 10^7^ Markov chain Monte Carlo (MCMC) steps (sampling every 5 × 10^3^ step) with an HKY+Γ substitution model and priors with low information content were set for the constant birth–death and coalescent exponential models (Table 1). However, for the birth death model δ was fixed to 36.5 years^−1^. Using priors with low information content allows us to evaluate the extent to which genetic variation in the data (e.g., number of variable sites) is sufficient to recover the set of epidemiological parameters used to simulate the datasets. TRACER v1.7.1 [23] was utilised to examine the results of the analyses and sufficient sampling was determined when key epidemiological parameters had effective sample size of at least 200.

Statistical analysis involved calculating the precision, coverage, and bias of our growth rate estimation from our analyses. The precision was the measure of the length of the 95% credible interval (CI), such that low numbers are improved precision. Calculating bias was a measure of the difference between the mean estimated value and the true value, which was also a measure of how accurate the results were, while the coverage was a measure of the number times the true value of a parameter of interest fell within the 95% CI, within set of simulations.

### 2.2. Empirical Data Analyses

Recent molecular datasets of SARS-CoV-2 outbreaks from Victoria and New Zealand were used to illustrate the results of our simulation study in real world data. The sequence data collected were from previous studies and obtained through GISAID [24] (acknowledgements table in Supplementary Materials). The Victorian outbreak dataset contained 92 whole genome samples from mid-March to early May. This dataset features a highly sampled outbreak cluster that was contained by the end of it. The New Zealand dataset contained 44 whole genome that were sampled from mid-March to early April. This cluster contained 41 sequences from New Zealand and 3 sequences from USA, the source of the most likely introduction into New Zealand. The cluster was intensely sampled throughout the outbreak and only the early exponential growth phase of the cluster was used in our phylodynamic analysis. Importantly, because our datasets consist of a short period of the SARS-CoV-2 pandemic, we expect a low number of substitutions.

These datasets were subjected to Bayesian phylodynamic analyses using BEAST2. The epidemiological models used to infer the epidemiological dynamics were the coalescent exponential-growth and the constant birth-death models. We assume a strict molecular clock under the HKY+Γ substitution model and the same priors as in our simulations. These analyses were then repeated while ignoring sequence data (i.e., “sampling from the prior”) and results were compared to those analyses with sequence data. Comparing the prior and posterior allows us to examine the contribution of the sequence data on the epidemiological parameter inferences.

## 3. Results

### 3.1. Simulation Study

Estimation of the growth rate and molecular clock rate were assessed in all the simulations and results from the coalescent exponential and constant rate birth–death models were compared. To assess the impact of the number of variable sites on estimating the true value of growth rate, the mean and 95% CI were also compared across the datasets. Statistical analysis on the model’s ability to re-capture true epidemiological parameters were performed and the bias, precision and coverage were recorded.

**Figure 1:**
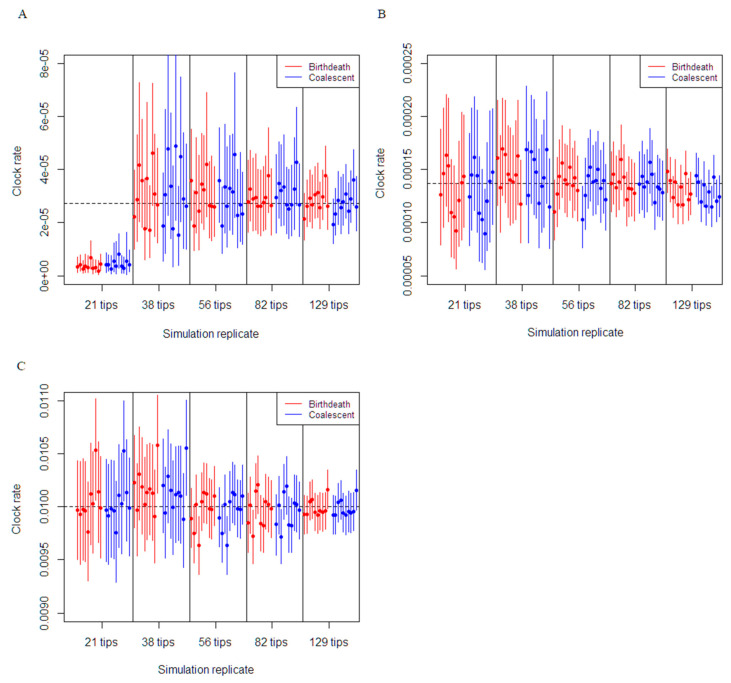
Comparison of birth–death and coalescent exponential models on molecular clock rate estimation for each simulation dataset. The plot depicts the 95% CI of molecular clock rate inferences from each dataset. The black dashed horizontal line depicts the true molecular clock rate used for the simulation. (**A**) Simulations set at the medium clock rate (0.001/36.5 subs/site/duration of infection). (**B**) Simulations set at five times the medium clock rate (0.005/36.5 subs/site/duration of infection). (**C**) Simulations set at a high clock rate (0.01 subs/site/duration of infection).

The molecular clock estimates were shown to be robust in the majority of the simulations for each clock rate setting. For each dataset with the clock rate set at 0.01 subs/site/duration of infection, which resulted in a substantial number of variable sites (i.e., between 3100 to 15,000), the true clock rate was estimated with accuracy and precision for both the coalescent exponential growth and constant birth–death model. For example, 93% of the analyses results from both models yielded true clock rate value within the 95% CI and with 95% CI widths decreasing from 0.0006 to 0.0002, as more sequences were added (Figure 1). Similarly, the estimation of molecular clock rate for datasets with the clock rate set at 0.005/36.5 subs/site/duration of infection (datasets with over 45 and up to 310 variable sites) were also highly precise and accurate, with 94% of simulations results from both models capturing true clock rate within the 95% CI and an average 95% CI width decreasing from 0.0006 to 0.0002, as more sequences were added.

In the phylogenetic tree simulations with 129 tips, 82 tips and 56 tips, the estimation of the clock rate parameter for 0.001/36.5 subs/site/duration of infection was accurate and the true rate was still within the 95% CI for both the coalescent and birth-death models (Figure 1A). Simulations using the phylogenetic tree with 38 tips were substantially less reliable with an average 95% CI width of 0.0004 for the coalescent and birth–death models. Finally, simulations using the 21-tip phylogenetic tree were unable to recapture the true value of the molecular clock rate for all the analyses. In regard to the effects of the number of variable sites, performance to recapture the true estimate of the molecular clock were relatively similar for the coalescent exponential growth and constant birth–death model.

Coverage of the growth rate parameter estimates were robust in the majority of the simulations with the clock rate set at 0.01 subs/site/duration of infection (Table 2). The phylodynamic analyses using the birth–death model with high number of tips (129 and 82) resulted in average relative bias of −0.145 for 129 tips and of −0.13 for 82 tips, causing slight underestimation of the true growth rate parameter. In addition, the growth rate parameter was accurately estimated for the trees with 56 and 21 tips, although the precision worsened with the mean 95% CI increasing from 1.51 to 2.66, as the number of tips decreased. Similarly, we found the same pattern using the coalescent model. The coalescent model estimation of growth rate parameter was accurate for the trees with (129, 82, 56, and 21) tips, while precision worsened from 0.58 to 1.47, as the number of tips decreased. The overall precision (narrow 95% CI) of the coalescent was better than for the birth–death model for each set of simulations. The simulations using the 38 tips tree resulted in an upward bias for both the coalescent and birth–death model, with an average value of 2 instead of 1.5.

**Figure 2:**
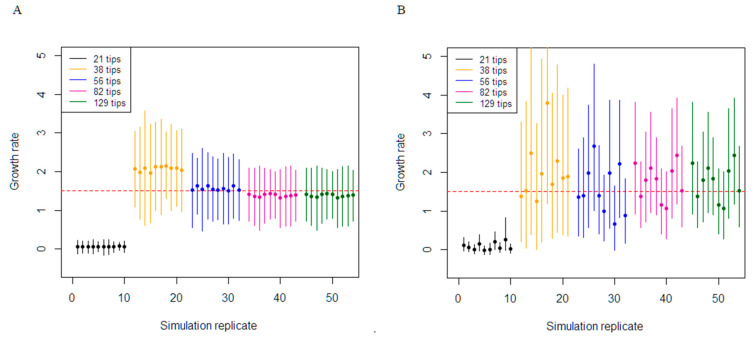
Comparison of the constant birth–death and coalescent exponential in estimating epidemic growth rate. The 95% highest posterior density results of growth rate re-estimation from the simulation set at the medium clock rate (0.001/36.5 subs/site/duration of infection). The different colour represents a simulation dataset with various number of tips. The mean of the posterior estimate of growth rate for each simulation is reported. The red horizontal line represents the true growth rate (1.5). (**A**) Depicts the results from the birth–death. (**B**) Depicts the results from the coalescent.

Inference performance for the medium substitution rate (0.001/36.5 subs/site/duration of infection) were dependent on the number of tips the phylogenetic tree contained to simulate the sequence data and by association the number of variable sites (Table 3 and Figure 2). The coverage of the true growth rate parameter for the birth–death analyses was 100% for the trees containing 129, 82, 56, and 38 tips. Although the coverage was 100%, there was a slight underestimation in the 129 simulations, with an average bias of −0.17. In addition, decreasing the number of variable sites dictated worse precision in the estimates, with precision worsening from 0.97 to 2.27. The growth-rate parameter for the coalescent exponential was highly variable in trees with 82, 56, and 38 tips, with very wide 95% CIs. For these analyses, the median growth rate estimates did not converge to a single value among datasets and were fluctuating between 0.65 to 3.78. The coverage for the coalescent was identical to the birth–death. Analyses from the 21-tips tree with the lowest number of variable sites failed to cover the true growth-rate in all the results and severely underestimated this parameter for the birth–death and coalescent model.

We obtained improved performance for simulation with the evolutionary rate set at 0.005/36.5 subs/site/duration of infection (Table 4). This clock rate setting increased variability among sequence data with an average of 45 for the 21-tip tree to 309 for the 129-tip tree. The coverage of the birth–death was 100% for all the analyses, whereas the coalescent was accurate for all but one analysis from the 56-tip tree simulations. The trend continues with high tip count resulting in improved precision, while precision worsens as the number of tips decreases. For example, the precision from high tip count to low, improved from 2.61 to 0.89 for the birth–death and from 2.64 to 0.89 for the coalescent. The birth–death slightly underestimated growth rate for the 129 and 82 simulations with a relatively bias of −0.17 and −0.11 respectively and the 21 and 38 tip tree resulted in a slight overestimation, with a relative bias of 0.11 and 0.31, respectively. Similarly, for the coalescent underestimation of growth rate occurred for the 129 and 56 tip tree with a relative bias of −0.11 and −0.13, respectively. Once again, the 21 and 38 tip tree subjected to the coalescent model resulted in mean growth rate was which was widely scattered across the simulations, however within the 95% CI.

Simulations with high number of tips (high sampling) produced estimations with high precision that always captured the correct parameter value, although occasionally with a slight bias towards underestimation. As the tip count decreases, the estimation converges towards the truth with worsening of precision. The number of tips for with lowest bias in estimation in our study was for the clock rate of 0.001/36.5 subs/site/duration for infection with 56 tips for both the coalescent and birth death model. This was also the case for the birth–death in the tree with the clock rate set at 0.005/36.5 subs/site/duration of infection, however the coalescent performed better with 82 tips. In terms of the number of variable sites, these results indicate as precision improves, biases are more likely to be observed, because the credible intervals are narrower.

### 3.2. Empirical Data Estimates of Molecular Clock Rate and Sampling

The Australian dataset from the state of Victoria analysis using the constant birth–death model estimated a molecular clock rate of 1.30 × 10^−3^ subs/site/year, with a 95% CI of 9.80 × 10^−4^ to 1.67 × 10^−3^ subs/site/year. Similarly, the coalescent exponential growth model inferred a mean molecular clock rate of 8.59 × 10^−4^ subs/site/year with a 95% CI from 3.86 × 10^−4^ to 1.37 × 10^−3^ subs/site/year. In addition to this, the molecular clock estimate using the New Zealand dataset were slightly higher using the birth–death model at 2.09 × 10^−3^ subs/site/year with 95% CI between 1.15 × 10^−3^ to 3.08 × 10^−3^ subs/site/year. This was also the case for the coalescent model estimation of clock rate was 3.28 × 10^−3^ with a CI range of 1.13 × 10^−3^ to 5.86 × 10^−3^ subs/site/year. These molecular clock rate estimates were consistent with results from previous studies using SARS-CoV-2 data [8,13], although those for the New Zealand data are on the upper range [25], which is expected due to their short sampling time window and potentially weak temporal signal, such that they should be interpreted with caution.

The empirical datasets used for our study were sequenced intensely during the outbreak. In Victoria, during the first wave, approximately 80% of COVID-19 cases were genetically sequenced [8]. Over in New Zealand, approximately 56% of the cases were sequenced [26]. Our sampling probability inferred by the birth–death model was 0.92 (CI: 0.77 to 1) for the Victorian cluster dataset and 0.85 (CI: 0.60:1) for the exponential growth phase of the New Zealand cluster. These estimates were in line with the sequencing efforts deployed in these locations.

### 3.3. Victorian Highly Sampled Outbreak Cluster Analysis

**Figure 3:**
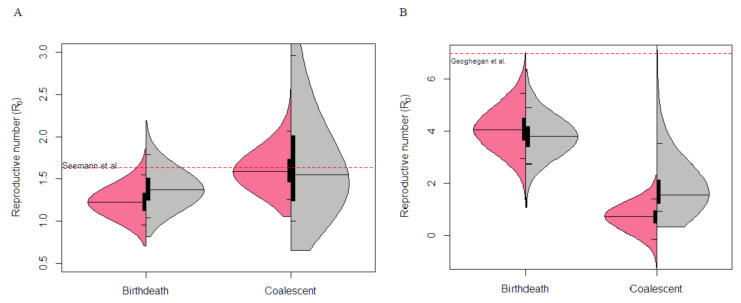
Basic Reproductive number, R_0_, estimation under the birth–death and coalescent model on empirical data. The posterior probability distribution of the R_0_ estimates with sequence data (pink) and without sequence data, i.e., “under the prior” (grey) is shown. The black bar indicates the interquartile range, the thin black lines indicate the 95% CI, while median is represented with the centre black line. The dashed red line represents estimates from analysis from previous studies. (**A**) Depicts the Australian cluster outbreak analysis with 92 samples and 174 variable sites. (**B**) Depicts the exponential phase of the New Zealand cluster outbreak with 44 samples and 28 variable sites. Note that previous estimates were conducted on the complete data set whereas here we considered the exponential growth phase only.

The Victorian dataset contained a highly sampled cluster outbreak of 92 samples and 174 variable sites among the sequences. Analysis using both the coalescent exponential and birth–death yielded similar inferences for the basic reproductive number (Figure 3). The birth–death model inferred a mean R_0_ value of 1.23 with a 95% CI between 0.94 and 1.52, furthermore the coalescent model inferred a mean R_0_ value of 1.61 with a CI of 1.22 to 2.03. Similar estimates were inferred from a dataset containing the whole outbreak (903 sequences) within Victoria [8].

### 3.4. New Zealand Exponential Cluster Analysis

The New Zealand dataset consist of 44 sequences and 28 variable sites that were acquired during the exponential growth phase of the outbreak cluster. The R_0_ estimates using the birth–death model was 4.10 (CI: 2.95 to 5.45). In contrast, the coalescent model inferred a R_0_ value of 0.68 (CI: −0.15 to 1.39) (Figure 3). Note that the lower bound of R_0_ falls below 0, which is an unusual pattern driven by a very low growth rate with a 95% CI of −41.78 to 14.13. The birth–death estimate were robust given similar results were recovered from a previous study using the birth–death skyline model estimating R_e_ changes over two-time intervals [26], although note that the previous study used the complete data set and fixed the clock rate, where as we estimated that parameter here. The R_e_ estimates before strict public health policies were introduced was seven (CI: 3.7–10.7), with our birth–death R_0_ estimate within the 95% CI. In contrast, the 95% CI of R_0_ estimate from the coalescent exponential-growth did not overlap and considerably underestimated R_0_ parameter. In both empirical analyses, the prior on R_0_ is very similar to the posterior in the birth–death model, reflecting the fact that this model can be largely informed by the sequence sampling times in addition to the sequence data [14].

## 4. Discussion

During emerging infectious disease outbreaks, it is crucial for epidemiologist to reliably quantify the spread within a population. The “phylodynamic threshold” is a relevant concept in our study because robust estimates of population dynamics are conditioned on allowing sufficient accumulation of genetic variation among sequence data to calibrate the molecular clock. Instead of testing the number of samples required, we explored the amount of genetic variation between sequence data required for robust estimates of epidemiological parameters from the coalescent exponential and birth–death model. This concept of measuring change within evolving populations has revolutionised our ability to study population dynamics through phylodynamic inference [27]. The information in sequence data determines two distinct parameters: the clock rate and tree topology and branch lengths [14].

Our results from our simulation study demonstrate accurate inference of molecular clock rates were possible when enough variability occurred between sequence data rather than the number of sequences. Accurate estimation of the molecular clock rate is conditioned on sufficient temporal signal from sequence data and is a necessary step for phylodynamic inference [28]. The simulations with sufficient evolutionary change between samples, i.e., clock rate set to 0.01 subs/site/duration of infection and 0.005/36.5 subs/site/duration of infection (Figure 1), models appeared to have strong temporal signal, producing accurate and precise estimates of the molecular clock rate. The number of sequences becomes the main factor in calibrating the molecular clock when there was insufficient sequence evolution. Our results supported this concept, with the datasets simulated with a clock rate set at 0.001/36.5 subs/site/duration of infection on the 38–129 tip trees, estimating the molecular clock rate reliably. This was not the case with the 21-tip tree, where the molecular clock rate was considerably underestimated. In comparison, accurate molecular clock estimation occurred on the same tree simulated with five times the medium rate of evolution. The “phylodynamic threshold” for the molecular clock was reached when adequate molecular evolution (18–25 variable sites) occurred within our monophyletic datasets and increasing the number of samples caused an improvement in precision.

In our simulation study, we generated trees under a birth–death process to demonstrate the stochastic nature of disease outbreak emergence and with different clock rates to exhibit how the number of variable sites affects phylodynamic inference. Under the coalescent model, the 95% CI of growth rate inferred was relatively narrow compared to the birth–death model. This results in better precision if thetrue growth rate was captured within this interval. The cause of this narrow CI intervals is primarily due to the deterministic nature of the coalescent model, where the coalescent only considers the exponential growing population trajectory while ignoring stochasticity [14]. In contrast, the results from the birth–death analyses tend to have a wider 95% CI around the growth rate. This is probably caused by the birth–death process accounting for stochasticity in the branching patterns, which leads to averaging out the epidemiological processes [29]. Our findings support this, as majority of our birth–death results estimated true growth rate with a wider 95% CI.

The birth–death model considers sampling as a parameter by modelling this process from the sampling times, whereas the coalescent model is conditioned on the sampling times on driving the effective population at any given time [9,29]. Phylodynamic inference using the birth–death model with sequence data with low diversification tend to outperform the coalescent exponential model in our simulation study. The 56 tip trees that were simulated with the medium clock rate (0.001/36.5 subs/site/duration of infection) resulted in sequences with only about 24 variable sites, where the birth–death gave more reliable estimates of the growth rate than the coalescent (Table 3). Our analyses with the birth–death and coalescent was not able to calibrate the molecular clock for the 21-tip tree, which is a necessity for phylodynamic inference if the molecular clock rate is not known a priori. However, there was enough sequence variability with the 38-tip tree dataset to calibrate the molecular clock for phylodynamic inference. From our findings, the birth–death analyses when faced with low variable data was able to estimate growth rate for the 38-tip tree, although with high uncertainty. This uncertainty decreases as additional sequences were added to the datasets, which also increased the intersequence variability.

The coalescent model on the other hand does not utilise the additional information provided from sampling times. This model only parameterises population size at any given time, which does not exploit the full extent of the epidemiological data [11]. Results from our simulation study using the coalescent model indicate poor performance in epidemiological parameter estimation in situations of low variability in sequence data. Although, the true growth rate from our analyses were within the 95% CI, the mean estimates for each individual simulation were fluctuating among each tree dataset (38, 56, and 82 tips) simulated with the medium clock rate (0.001/36.5 subs/site/duration of infection). Our simulation results revealed that the coalescent model requires further samples and therefore increased variability between sequences to accurately infer epidemiological parameters, in contrast to the birth–death model.

The simulations conducted in our study used a birth–death process which generated a closely related group of sequences, to resemble a cluster outbreak and served as a proof of concept when analysing empirical data. The highly sampled Victorian dataset contained a sufficient number of samples and sequence diversity and phylodynamic analysis using either the coalescent or birth–death model yields robust estimates of epidemiological parameters. Our results from our study were R_0_ values of 1.23 (birth–death) and 1.61 (coalescent), which aligned with similar R_0_ estimate with informative prior selections from a birth–death skyline model.

Phylodynamic analysis on the exponential growth phase of the New Zealand cluster (44 samples/28 variable sites), the birth–death model was able to infer reasonable estimates (R_0_ = 4.10, CI: 2.95 to 5.45) of epidemiological parameters, which was in line with estimates from a previous study [26]. In contrast the coalescent significantly underestimated the R_0_ parameter (0.68, CI: −0.15 to 1.39), despite obtaining a reasonable clock rate, suggesting temporal signal in the data. The coalescent model makes the assumption that the number of samples must be much lower than the effective population size, which exposes the disadvantage of the coalescent to adapt to stochastic population growth in the data [10].

A common practice in Bayesian phylodynamic inference is to run these analyses “under the prior” to reveal the amount of information in sequence data that contributes to the estimates, compared to the prior assumptions and underlying model. This technique may evaluate if there is enough information in the sequences for phylodynamic inference. In addition, model adequacy methods involves evaluating the absolute model fit that best describes the data, which could be used for model selection during epidemic investigations [7]. This technique enables researchers to recognise if the model selected is a good fit for the data. However, model adequacy methods are severely underdeveloped in phylodynamics [30].

Overall, when performing Bayesian phylodynamic analyses during emerging epidemics, researchers should take careful consideration in selecting the model. Our findings suggest the birth–death model is more robust when faced with data with low sequence diversity, given that the sampling process is correctly specified, unlike the coalescent model which required considerably more intersequence variability to improve performance [31]. A limitation of our study is that we generated a phylogenetic tree representing a cluster event with constant sampling over time and our empirical data analyses were also intensely sampled over time within a single cluster. Sequence data with irregular sampling patterns may result in the birth–death model inferring biased epidemiological parameters. Future studies may consider the effects of phylodynamic inference using sequence data with low variability and irregular sampling patterns and/or homochromous sampling. In addition, future studies should assess the impact of low sequence variation in more complex epidemiological models. Evaluating these phylodynamic methods is essential for future model uptake in epidemic investigations.

## Figures and Tables

**Table 1:** Overview of models, parameters, and priors.

Phylodynamic Model	Parameter	Value	Substitution Model	Clock Model
Constant rate birth–death	Effective reproductive number (R_0_)	Estimated. Prior; Log-normal distribution with mean = 0, sigma = 1	HKY+Γ	Strict clock
	Become uninfectious rate (δ)	Fixed; 36.5 years^−1^		
	Sampling probability (p)	Estimated. Prior; Beta distribution with (α and β) = 1		
Coalescent exponential	Effective population size (N_e_)	Estimated. Prior; Log-normal distribution with mean = 1, sigma = 2	HKY+Γ	Strict clock
	Growth rate (r)	Estimated. Prior; Laplace distribution with μ = 0, scale = 30.70		

**Table 2:** Simulation results from clock rate set at the large accumulation of variability (0.01 subs/site/duration of infection) for constant rate birth–death and coalescent exponential growth model. Good precision corresponds to low relative precision values.

	Dataset (Samples)	Variable Sites (Mean)	Coverage (%)	Relative Bias	Relative Precision (95% CI Width)
Birth–death estimation of growth rate (Truth—1.5)	21 tips	3154	100	0.07	2.66
	38 tips	4857	100	0.21	2.19
	56 tips	7875	100	0.01	1.51
	82 tips	9956	100	−0.13	1.32
	129 tips	15537	100	−0.15	0.93
Coalescent estimation of growth rate (Truth—1.5)	21 tips	3154	100	−0.05	1.47
	38 tips	4857	100	0.12	1.72
	56 tips	7875	100	−0.07	0.89
	82 tips	9956	100	−0.02	0.83
	129 tips	15537	100	−0.09	0.58

Note—The mean of all bias/precision result for each simulation setting is provided.

**Table 3:** Simulation results from clock rate set at the medium rate (0.001/36.5 subs/site/duration of infection) for constant rate birth–death and coalescent exponential growth model. Good precision corresponds to low relative precision values.

	Dataset	Variable Sites (Mean)	Coverage (%)	Relative Bias	Relative Precision (95% CI Width)
Birth–death estimation of growth rate (Truth—1.5)	21 tips	9	0	−0.96	0.31
	38 tips	16	100	0.39	2.27
	56 tips	24	100	0.04	1.73
	82 tips	34	100	−0.08	1.44
	129 tips	62	100	−0.17	0.97
Coalescent estimation of growth rate (Truth—1.5)	21 tips	9	0	−0.94	0.36
	38 tips	16	100	0.34	4.14
	56 tips	24	100	0.04	2.55
	82 tips	34	100	0.17	2.26
	129 tips	62	100	−0.08	1.42

Note—The mean of all bias/precision result for each simulation setting is provided.

**Table 4:** Simulation results from clock rate set at five times the medium rate (0.005/36.5 subs/site/duration of infection) for constant rate birth–death and coalescent exponential growth model. Good precision corresponds to low relative precision values.

	Dataset	Variable Sites (Mean)	Coverage (%)	Relative Bias	Relative Precision (95% CI Width)
Birth–death estimation of growth rate (Truth—1.5)	21 tips	45	100	0.11	2.61
	38 tips	72	100	0.31	2.41
	56 tips	127	100	0.00	1.53
	82 tips	158	100	−0.11	1.40
	129 tips	309	100	−0.17	0.89
Coalescent estimation of growth rate (Truth—1.5)	21 tips	45	100	0.18	2.52
	38 tips	72	100	0.26	2.64
	56 tips	127	90	−0.13	1.28
	82 tips	158	100	−0.01	1.23
	129 tips	309	100	−0.11	0.80

Note—The mean of all bias/precision result for each simulation setting is provided.

## Data Availability

The data presented in this study are openly available in the GISAID and all sequence accession numbers are listed in the Supplementary Materials (Table S1).

## Supplementary Materials

The following are available online at https://www.mdpi.com/1999-4915/13/1/79/s1, Table S1. Accession numbers and GISAID labels for sequences used in our empirical analyses.
