# Supplementary material for: The Impacts of Low Diversity Sequence Data on Phylodynamic Inference during an Emerging Epidemic
Source: Viruses. 2021 Jan 8;13(1):79. doi: 10.3390/v13010079 (PMC7826997; doi:10.3390/v13010079)
Supplement: Supplementary file 1 [file viruses-13-00079-s001.pdf]

We gratefully acknowledge the following Authors from the Originating laboratories responsible for obtaining the specimens and the Submitting laboratories where genetic sequence data were generated and shared via the GISAID Initiative, on which this research is based.

Virus name	Accession No.	Collected	Originating laboratory	Submitting laboratory	Authors
hCoV-19/Australia/VIC319/2020	EPI_ISL_420010	2020-03-24	Microbiological Diagnostic Unit Public Health Laboratory	Microbiological Diagnostic Unit Public Health Laboratory	Seemann T., Schultz M., Sait, M., Sherry, N.
hCoV-19/Australia/VIC320/2020	EPI_ISL_420011	2020-03-24			
hCoV-19/Australia/VIC964/2020	EPI_ISL_430655	2020-03-27			
hCoV-19/Australia/VIC974/2020	EPI_ISL_430659	2020-03-28			
hCoV-19/Australia/VIC967/2020	EPI_ISL_430660	2020-03-27			
hCoV-19/Australia/VIC476/2020	EPI_ISL_426908	2020-03-28			
hCoV-19/Australia/VIC479/2020	EPI_ISL_426911	2020-03-28			
hCoV-19/Australia/VIC485/2020	EPI_ISL_426916	2020-03-29			
hCoV-19/Australia/VIC486/2020	EPI_ISL_426917	2020-03-29			
hCoV-19/Australia/VIC781/2020	EPI_ISL_427056	2020-03-30			
hCoV-19/Australia/VIC783/2020	EPI_ISL_427058	2020-03-30			
hCoV-19/Australia/VIC785/2020	EPI_ISL_427060	2020-03-30			
hCoV-19/Australia/VIC787/2020	EPI_ISL_427062	2020-03-30			
hCoV-19/Australia/VIC793/2020	EPI_ISL_427068	2020-03-31			
hCoV-19/Australia/VIC797/2020	EPI_ISL_427071	2020-04-01			
hCoV-19/Australia/VIC800/2020	EPI_ISL_427074	2020-04-01			
hCoV-19/Australia/VIC2075/2020	EPI_ISL_521910	2020-04-01			
hCoV-19/Australia/VIC804/2020	EPI_ISL_427078	2020-04-01			
hCoV-19/Australia/VIC2076/2020	EPI_ISL_480616	2020-04-02			
hCoV-19/Australia/VIC1030/2020	EPI_ISL_430664	2020-04-02			
hCoV-19/Australia/VIC1032/2020	EPI_ISL_430665	2020-04-02			
hCoV-19/Australia/VIC1054/2020	EPI_ISL_430666	2020-04-03			
hCoV-19/Australia/VIC1068/2020	EPI_ISL_430671	2020-04-04			
hCoV-19/Australia/VIC1064/2020	EPI_ISL_430672	2020-04-04			
hCoV-19/Australia/VIC898/2020	EPI_ISL_427148	2020-04-08			
hCoV-19/Australia/VIC399/2020	EPI_ISL_426702	2020-03-24	Victorian Infectious Diseases Reference Laboratory (VIDRL)	Microbiological Diagnostic Unit Public Health Laboratory and Victorian Infectious Diseases Reference Laboratory, Doherty Institute	Caly L., Seemann T., Sait, M., Schultz M., Druce J., Sherry, N.
hCoV-19/Australia/VIC541/2020	EPI_ISL_426806	2020-03-24			
hCoV-19/Australia/VIC557/2020	EPI_ISL_426818	2020-03-25			
hCoV-19/Australia/VIC563/2020	EPI_ISL_426824	2020-03-24			
hCoV-19/Australia/VIC417/2020	EPI_ISL_426717	2020-03-25			
hCoV-19/Australia/VIC419/2020	EPI_ISL_426719	2020-03-25			
hCoV-19/Australia/VIC460/2020	EPI_ISL_426753	2020-03-26			
hCoV-19/Australia/VIC465/2020	EPI_ISL_426758	2020-03-27			

hCoV-19/Australia/VIC610/2020	EPI_ISL_426864	2020-03-26			
hCoV-19/Australia/VIC628/2020	EPI_ISL_426932	2020-03-29			
hCoV-19/Australia/VIC635/2020	EPI_ISL_426876	2020-03-28			
hCoV-19/Australia/VIC637/2020	EPI_ISL_426933	2020-03-28			
hCoV-19/Australia/VIC682/2020	EPI_ISL_426977	2020-03-29			
hCoV-19/Australia/VIC708/2020	EPI_ISL_426995	2020-03-29			
hCoV-19/Australia/VIC718/2020	EPI_ISL_427002	2020-03-27			
hCoV-19/Australia/VIC920/2020	EPI_ISL_427153	2020-03-27			
hCoV-19/Australia/VIC760/2020	EPI_ISL_427036	2020-03-29			
hCoV-19/Australia/VIC991/2020	EPI_ISL_430474	2020-03-31			
hCoV-19/Australia/VIC1001/2020	EPI_ISL_430485	2020-03-31			
hCoV-19/Australia/VIC1002/2020	EPI_ISL_430486	2020-03-31			
hCoV-19/Australia/VIC1004/2020	EPI_ISL_430488	2020-03-31			
hCoV-19/Australia/VIC1197/2020	EPI_ISL_430506	2020-03-31			
hCoV-19/Australia/VIC1236/2020	EPI_ISL_430524	2020-04-02			
hCoV-19/Australia/VIC1024/2020	EPI_ISL_430531	2020-04-02			
hCoV-19/Australia/VIC1025/2020	EPI_ISL_430532	2020-04-02			
hCoV-19/Australia/VIC1046/2020	EPI_ISL_430550	2020-04-03			
hCoV-19/Australia/VIC989/2020	EPI_ISL_430551	2020-03-31			
hCoV-19/Australia/VIC816/2020	EPI_ISL_427089	2020-04-03			
hCoV-19/Australia/VIC843/2020	EPI_ISL_427103	2020-04-04			
hCoV-19/Australia/VIC853/2020	EPI_ISL_427113	2020-04-04			
hCoV-19/Australia/VIC870/2020	EPI_ISL_427125	2020-04-05			
hCoV-19/Australia/VIC885/2020	EPI_ISL_427138	2020-04-06			
hCoV-19/Australia/VIC1047/2020	EPI_ISL_430561	2020-04-03			
hCoV-19/Australia/VIC1048/2020	EPI_ISL_430562	2020-04-03			
hCoV-19/Australia/VIC1097/2020	EPI_ISL_521865	2020-04-07			
hCoV-19/Australia/VIC1131/2020	EPI_ISL_430592	2020-04-08			
hCoV-19/Australia/VIC1086/2020	EPI_ISL_430597	2020-04-06			
hCoV-19/Australia/VIC1157/2020	EPI_ISL_430598	2020-04-09			
hCoV-19/Australia/VIC1050/2020	EPI_ISL_430599	2020-04-03			
hCoV-19/Australia/VIC1134/2020	EPI_ISL_430600	2020-04-08			
hCoV-19/Australia/VIC1141/2020	EPI_ISL_430612	2020-04-08			
hCoV-19/Australia/VIC1328/2020	EPI_ISL_430695	2020-04-12			
hCoV-19/Australia/VIC1285/2020	EPI_ISL_430702	2020-04-09			
hCoV-19/Australia/VIC1375/2020	EPI_ISL_456416	2020-04-14			
hCoV-19/Australia/VIC1410/2020	EPI_ISL_456429	2020-04-16			
hCoV-19/Australia/VIC1412/2020	EPI_ISL_456431	2020-04-16			
hCoV-19/Australia/VIC1882/2020	EPI_ISL_480592	2020-04-16			

hCoV-19/Australia/VIC1428/2020<br>hCoV-19/Australia/VIC1432/2020<br>hCoV-19/Australia/VIC1438/2020<br>hCoV-19/Australia/VIC1441/2020<br>hCoV-19/Australia/VIC1464/2020<br>hCoV-19/Australia/VIC1465/2020<br>hCoV-19/Australia/VIC1459/2020<br>hCoV-19/Australia/VIC1486/2020<br>hCoV-19/Australia/VIC1495/2020<br>hCoV-19/Australia/VIC1501/2020<br>hCoV-19/Australia/VIC1572/2020<br>hCoV-19/Australia/VIC1996/2020<br>hCoV-19/Australia/VIC192/2020<br>hCoV-19/Australia/VIC217/2020<br>hCoV-19/Australia/VIC224/2020<br>hCoV-19/Australia/VIC266/2020<br>hCoV-19/Australia/VIC270/2020<br>hCoV-19/Australia/VIC271/2020	EPI_ISL_456435<br>EPI_ISL_456439<br>EPI_ISL_456442<br>EPI_ISL_456443<br>EPI_ISL_456446<br>EPI_ISL_456447<br>EPI_ISL_456448<br>EPI_ISL_456455<br>EPI_ISL_456459<br>EPI_ISL_456466<br>EPI_ISL_456489<br>EPI_ISL_521902<br>EPI_ISL_419888<br>EPI_ISL_419912<br>EPI_ISL_419919<br>EPI_ISL_419959<br>EPI_ISL_419963<br>EPI_ISL_419964	2020-04-17<br>2020-04-17<br>2020-04-18<br>2020-04-19<br>2020-04-21<br>2020-04-22<br>2020-04-21<br>2020-04-25<br>2020-04-29<br>2020-04-27<br>2020-05-07<br>2020-03-26<br>2020-03-19<br>2020-03-20<br>2020-03-20<br>2020-03-22<br>2020-03-22<br>2020-03-22			
hCoV-19/New Zealand/20VR1615/2020<br>hCoV-19/New Zealand/20VR1777/2020<br>hCoV-19/New Zealand/20VR1778/2020<br>hCoV-19/New Zealand/20VR1809/2020<br>hCoV-19/New Zealand/20VR1814/2020<br>hCoV-19/New Zealand/20VR1816/2020<br>hCoV-19/New Zealand/20VR1817/2020<br>hCoV-19/New Zealand/20VR1818/2020<br>hCoV-19/New Zealand/20VR1823/2020<br>hCoV-19/New Zealand/20VR1825/2020<br>hCoV-19/New Zealand/20VR1827/2020<br>hCoV-19/New Zealand/20VR1838/2020<br>hCoV-19/New Zealand/20VR1822/2020<br>hCoV-19/New Zealand/20VR1826/2020<br>hCoV-19/New Zealand/20VR1828/2020<br>hCoV-19/New Zealand/20VR1845/2020<br>hCoV-19/New Zealand/20VR1843/2020<br>hCoV-19/New Zealand/20VR1821/2020	EPI_ISL_456225<br>EPI_ISL_456246<br>EPI_ISL_456247<br>EPI_ISL_456261<br>EPI_ISL_456262<br>EPI_ISL_456263<br>EPI_ISL_456264<br>EPI_ISL_456265<br>EPI_ISL_456268<br>EPI_ISL_456270<br>EPI_ISL_456272<br>EPI_ISL_456279<br>EPI_ISL_456267<br>EPI_ISL_456271<br>EPI_ISL_456273<br>EPI_ISL_456282<br>EPI_ISL_456281<br>EPI_ISL_456266	2020-03-24<br>2020-03-26<br>2020-03-25<br>2020-03-27<br>2020-03-27<br>2020-03-27<br>2020-03-27<br>2020-03-27<br>2020-03-27<br>2020-03-27<br>2020-03-27<br>2020-03-28<br>2020-03-27<br>2020-03-27<br>2020-03-27<br>2020-03-29<br>2020-03-28<br>2020-03-27	Southern Community Labs Dunedin	Institute of Environmental Science and Research (ESR)	Matt Storey, Xiaoyun Ren, Anja Werno, Antje van der Linden, Arlo Upton, Chris Mansell, David Hammer, Dragana Drinkovic, Erasmus Smit, Gary McAuliffe, Hana Sofia Andersson, James Ussher, Jill Sherwood, Josh Freeman, Julia Howard, Juliet Elvy, Mary DeAlmeida, Matt Blakiston, Matthew Rogers, Max Bloomfield, Michael Addidle, Michelle Balm, Sally Roberts, Sarah Jefferies, Sharmini Muttaiyah, Susan Morpeth, Susan Taylor, Timothy Blackmore, Vani Sathyendran, Veronica Playle, Virginia Hope, Erasmus Smit, Lauren Jelly, Joep de Ligt

hCoV-19/New Zealand/20VR2002/2020<br>hCoV-19/New Zealand/20VR1997/2020<br>hCoV-19/New Zealand/20VR1990/2020<br>hCoV-19/New Zealand/20VR2001/2020	EPI_ISL_456329<br>EPI_ISL_456326<br>EPI_ISL_456324<br>EPI_ISL_456328	2020-04-01<br>2020-04-01<br>2020-04-01<br>2020-04-01	Wellington SCL		
hCoV-19/New Zealand/20VR2522/2020	EPI_ISL_456382	2020-04-15	LabPLUS		
hCoV-19/New Zealand/20VR1775/2020<br>hCoV-19/New Zealand/20VR1812/2020<br>hCoV-19/New Zealand/20VR1820/2020	EPI_ISL_579128<br>EPI_ISL_579137<br>EPI_ISL_579141	2020-03-25<br>2020-03-28<br>2020-03-27	Southern Community Labs Dunedin	Institute of Environmental Science and Research (ESR)	Xiaoyun Ren, Matt Storey, Nikki Freed, Muhammad Faisal, Jing Wang, Hermes Perez, Anja Werno, Antje van der Linden, Arlo Upton, Chris Mansell, David Hammer, Dragana Drinkovic, Gary McAuliffe, Hana Sofia Andersson, James Ussher, Jill Sherwood, Josh Freeman, Julia Howard, Juliet Elvy, Mary DeAlmeida, Matt Blakiston, Matthew Rogers, Max Bloomfield, Michael Addidle, Michelle Balm, Sally Roberts, Sarah Jefferies, Sharmini Muttaiyah, Susan Morpeth, Susan Taylor, Timothy Blackmore, Vani Sathyendran, Veronica Playle, Virginia Hope, Erasmus Smit, Lauren Jelly, Olin Silander, Joep de Ligt
hCoV-19/New Zealand/20VR1993/2020<br>hCoV-19/New Zealand/20VR1999/2020<br>hCoV-19/New Zealand/20VR3026/2020<br>hCoV-19/New Zealand/20VR3022/2020<br>hCoV-19/New Zealand/20VR1988/2020	EPI_ISL_579194<br>EPI_ISL_579198<br>EPI_ISL_579392<br>EPI_ISL_579389<br>EPI_ISL_579191	2020<br>2020<br>2020<br>2020<br>2020	Wellington SCL (WN)		
hCoV-19/New Zealand/20VR2919/2020<br>hCoV-19/New Zealand/20VR2486/2020<br>hCoV-19/New Zealand/20VR2497/2020<br>hCoV-19/New Zealand/20VR2647/2020<br>hCoV-19/New Zealand/20VR2537/2020<br>hCoV-19/New Zealand/20VR2948/2020	EPI_ISL_579350<br>EPI_ISL_579243<br>EPI_ISL_579252<br>EPI_ISL_579315<br>EPI_ISL_579282<br>EPI_ISL_579368	2020<br>2020<br>2020<br>2020<br>2020<br>2020	LabPLUS		
hCoV-19/New Zealand/20VR3182/2020<br>hCoV-19/New Zealand/20VR3196/2020	EPI_ISL_579470<br>EPI_ISL_579482	2020-03-25<br>2020-03-27	Canterbury Health Laboratories		
hCoV-19/USA/NY-PV08120/2020<br>hCoV-19/USA/NY-PV09141/2020<br>hCoV-19/USA/NY-PV09328/2020	EPI_ISL_421349<br>EPI_ISL_422512<br>EPI_ISL_450017	2020-03-17<br>2020-03-20<br>2020-03-18	MSHS Clinical Microbiology Laboratories	MSHS Pathogen Surveillance Program	Ana S. Gonzalez-Reiche, Mitchell Sullivan, Ajay Obla, Gopi Patel, Emilia Sordillo, Melissa Gitman, Alberto Paniz-mondolfi, Matthew

					Hernandez, Shelcie Fabre, Jose Polanco, Zenab Khan, Bremy Albuquerque, Jayeeta Dutta, Juan Soto, Shwetha Sridhar Hara, Ying-Chih Wang, Melissa Smith, Robert Sebra, Lisa Miorin, Wen-chun Liu, Randy Albrecht, Judith Aberg, Florian Krammer, Adolfo Garcia-Sarstre, Viviana Simon, Harm van Bakel
